# PD32 - Non-immediate type beta-lactam allergy in childhood

**DOI:** 10.1186/2045-7022-4-S1-P32

**Published:** 2014-02-28

**Authors:** Ozlem Cavkaytar, Ozge Uysal Soyer, Betul Buyuktiryaki, Ebru Arik Yilmaz, Cansin Sackesen, Bulent E Sekerel, Ayfer Tuncer

**Affiliations:** 1 Department of Pediatric Allergy, Hacettepe University Medical Faculty, Ankara, Turkey

## Introduction

Even though non-immediate type allergic reactions with beta-lactam antibiotics occur more frequently than immediate reactions little is known about the actual prevalence of non-immediate beta-lactam allergy in childhood.

## Objective

To determine the rate and risk factors for non-immediate type beta-lactam allergy in children with a history of allergic reaction occurring at least 1 hour after the drug intake.

## Material and method

A prospective study was performed using ENDA questionnaire in 103 children [4.8 (2.7-7.9) years, 60% male] with 124 non-immediate type hypersensitivity reactions. Prick and intradermal skin testing with major/minor determinant mixture, penicilin G, ampicillin, amoxicillin-clavulanate and the culprit drug were done. In patients with negative skin tests, patch and intradermal tests with late readings were done with the culprit drug. Drug provocation was performed in case of negative interventional tests.

## Results

Fifteen children (14.6%) with 16 non-immediate reactions (12.9%) was diagnosed as beta-lactam allergic. The drugs responsible for non-immediate type beta-lactam allergy were amoxicillin-clavulanate (75%), sulbactam-ampicillin (6.3%), penicillin (6.3%), ceftriaxone (6.3%), cefaclor (6.3%).

The diagnosis of non-immediate type beta-lactam allergy was determined by provocation tests (n=11) prick/epidermal tests (n=3), patch tests (n=2). Only angioedema was found to increase the risk for diagnosis of non-immediate type beta-lactam allergy (OR:3.8, 95%CI:1.2-11.4, p=0.009).

## Conclusion

Diagnosis of drug allergy is also common in children with a history of nonimmediate reactions to beta-lactams. Classification due to timing of the reaction might not be appropriate to define the underlying pathogenesis of beta-lactam hypersensitivity since three patients had a positive skin test indicating IgE mediated beta-lactam allergy.Intradermal tests with late readings might not be practical in the diagnosis of non-immediate type beta-lactam allergy.

